# Molecular diagnosis of the human immunodeficiency, Hepatitis B and C viruses among blood donors in Lomé (Togo) by multiplex real time PCR

**DOI:** 10.11604/pamj.2016.25.242.7096

**Published:** 2016-12-20

**Authors:** Maléki Assih, Lochina Feteke, Cyrille Bisseye, Djeneba Ouermi, Florencia Djigma, Simplice Damintoti Karou, Jacques Simpore

**Affiliations:** 1 Center for Biomolecular Research Pietro Annigoni, CERBA/LABIOGENE, University of Ouagadougou, Burkina Faso; 2 National Center for Blood Transfusion (CNTS), Lomé, Togo; 3 Laboratory of Molecular and Cellular Biology, University of Sciences and Techniques of Masuku (USTM), Franceville, Gabon; 4 High School of Food and Biological Techniques (ESTBA-UL), University of Lomé, Togo

**Keywords:** Multiplex real time PCR, HBV, HCV, HIV

## Abstract

This study aimed to compare the sensitivity of multiplex PCR to ELISA technique in the instantaneous detection of HBV, HCV and HIVin blood samples from donors of the National blood Transfusion Centre in Togo. A total of 440 blood samplesfrom volunteer were collected and tested by ELISA and multiplex PCR for HBV, HCV and HIV detection. Among the 440 volunteer blood donors, 83% were female and 17% were male. Age range of 20-29 years was more represented (73%). Whereas, multiplex PCR detected more cases of HBV than ELISA (50% vs 33%, P=0.0155);ELISA more detected HCV than PCR (34% vs 3%, P<0.0001) and HIV (26% vs 7%, P<0.0001). Confirming these observations our data showed that multiplex PCR was more sensitive in the detection of HBV. The sensitivity of ELISA for the detection of HCV and HIV was elevated compared to multiplex PCR. Multiplex PCR was more specific that ELISA for the detection of HCV and HIV.Interestingly, our data showed that the gender do not influenced the sensitivity of either ELISA or multiplex PCR to detect these viruses. This study showed the limit of both ELISA and multiplex PCR in the detection of HBV, HCV and HIV.

## Introduction

The persistence of the risk of viral infection transmission in blood transfusion is a serious public health problem. To improve the quality of blood products (PSL) and ensure blood safety, a systematic serological screening of Human Immuno deficiency Virus (HIV), Hepatitis B virus (HBV), and Hepatitis C Virus (HCV)were introduced in the selection of blood donors [1–3]. In 2009, the prevalence of these viruses in Togo as reported by the National Blood Transfusion Center were 1.15% for HIV, 4.70% for HBV and 2.52% for HCV [4]. In the same year, in Burkina Faso, this prevalence was 2.21%; 14.96%and8.69% respectively for HIV, HBV and HCV, according to the report in blood donors in Koudougou [5]. In South Africa, prevalence of73.9%and89.4%were reported respectively for HBV and HIV among first blood donors [6]. Despite the measures taken to reduce or eliminate the transmission of these viruses by blood transfusion, the residual post-transfusion risk remains [7]. This persistence is related to the serological box during which the serological techniques are less effective. For this reason, a good transfusion policy based on rigorous screening strategies must be implemented [8]. The molecular methods such as viral genomic diagnosis (DGV) using molecular techniques such as the Polymerase Chain Reaction (PCR) in real time, applied to the detection and quantification of viral genomes, are more sensitive and do not expose to false positives associated with cross-contamination [9]. Indeed, a study on the characterization of the genotype of HCV among 2200 blood donors in the regional blood transfusion center of Ouagadougou in Burkina Faso in 2011 showed the prevalence of HCV antibodies and viral RNA respectively 4.4% and 1.5% by ELISA and PCR [10]. Using the PCR in the detection of the viral genome (DGV) has the advantage of reducing the silent phase of the most sought virus in blood donors. It can be fully automated, and considerably reduce the analysis duration [11]. In Togo, as in almost sub-Saharan Africa, due to the limited financial resources, the DGV is not yet introduced in blood transfusion. In this study we evaluated the effectiveness of multiplex real-time PCR, that can simultaneously detect the three major viruses (HBV, HCV and HIV) diagnosed among blood donors of the National Blood Transfusion Centre of Lomé.

## Methods

### Study population

Voluntary blood donors aged from 18 to 53 years were recruited according to the standard procedure of the National Blood Transfusion Centre of Lomé (Togo). These donors were all apparently healthy subjects selected after answering a questionnaire including their medical history and the risks of viral infections transmission. In total 440 samples from the volunteers, positive to one of the three viruses (HBV, HCV and HIV) using the fourth generation ELISA were collected. The Tests MonolisaTMHbs AgULTRA (Bio-Rad, France), Monolisa TMHCVAg-AbULTRA (Bio-Rad, France) and Genscreen TMHIVAg-AbULTRA (Bio-Rad, France) were used respectively for the serological screening of HBV, HCV and HIV.

### Nucleic acids extraction and real-time multiplex PCR

The DNA and RNA were extracted from 200µl of serum using Genomic Column DNA Express kit (Sacace Biotechnology, Como Italy) according to manufacturer´s instructions. This method of extracting the nucleicacids using a column is based on their adsorption on a silicate membrane contained in a column. After the wash the can be eluted with an elution solution provided by the manufacturer. For the amplification by multiplex real time PCR, a reaction volume of 25µl (mixture of 15µl of the extract product and 10µl of the reaction mix prepared from amplification kit, (kitHCV/HBV/HIV Real-TM, Sacace Biotechnologies, Como, Italy) was introduced in 96-well plates that were placed in PCR thermocycler 7500 Fast real Time PCR (Applied Biosystems, CA, USA). The following program was run: one step of 50°C for 20 minutes and 95 °C for 15 minutes and 45 cycles of 95 °C for 15 seconds, 60°C for 40 seconds and 45°C for 40 seconds. The HCV cDNA was detected in the AFM, the HIV cDNA was detected in the JOE and HBV DNA in the Texas Red.

### Statistical analyses

Data were recorded with the Microsoft Excel 2010 version. Statistical analyzes were performed with PRISM 5 (Graph Pad Software, Inc., La Jolla, USA). For the comparison χ2 test was performed using two-side Fisher’s exact test. The statistical threshold was defined as P ≤ 0.05.

## Results

**Study population characteristic:** in order to compare the sensitivity of ELISA and multiplex PCR in the detection of HBV, HCV and HIV we collected sera from 440 donors aged from 18-53years. Table 1 shows the characteristics of the study population. Men were more represented than women (82.90% vs 17.10%). The study population could be divided into four age groups as shown in the table. Among them, the age range from 20-29 years was significantly represented than the other groups. With regards to the professional occupation of the donors, students were also more represented compared to people working in the formal sector and in informal sector. Overall, 326 donors were at their first donation while 114 regularly donated the blood.

**Table 1:** Socio demographic profile of the study population

Characteristics		Number	Percentage
Sex	Male	365	82.90
	Female	75	17.10
Age group	< 20 years	54	12.30
	[20-29 years]	320	72.60
	[30-39 years]	54	12.30
	≥40 years	12	2.70
Occupation	Student	338	76.70
	Formal sector	33	7.50
	Informal sector	69	15.80
Type of donor	First donation	326	74.00
	Regular donation	114	26.00

**Figure 1:**
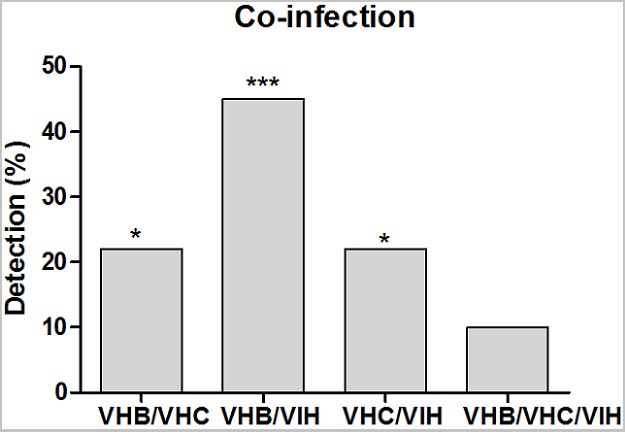
Frequencies of the co-infection

**Frequency of HBV and HIV infection:** four hundred forty (440) sera were screened by ELISA for the detection of the co-infection of the three viruses. Figure 1 indicates the co-infection rates between HBV/HCV, HBV/HIV, HCV/VIH and HBV/HCV/HIV. According to the figure, the co-infection HBV/HIV was more encountered, in more than 45%. This was followed in the order of importance by the co-infections HBV/HCV and HCV/VIH, rates under 30% and no statistically difference was observed between these frequencies. Furthermore, the triple co-infection HBV/HCV/HIV was detected in approximately 10% cases.

**Figure 2:**
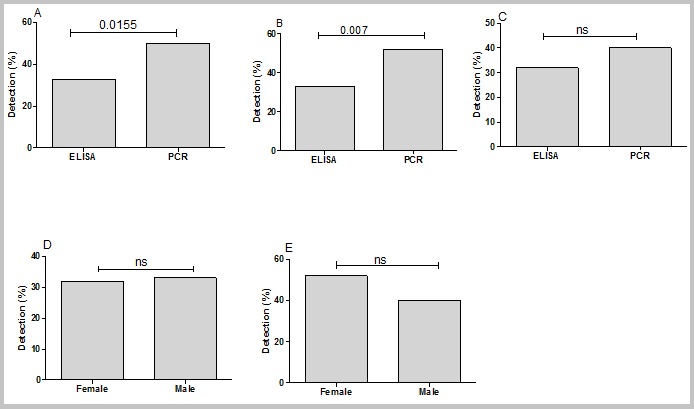
Detection of HBV by multiplex PCR and ELISA

**Figure 3:**
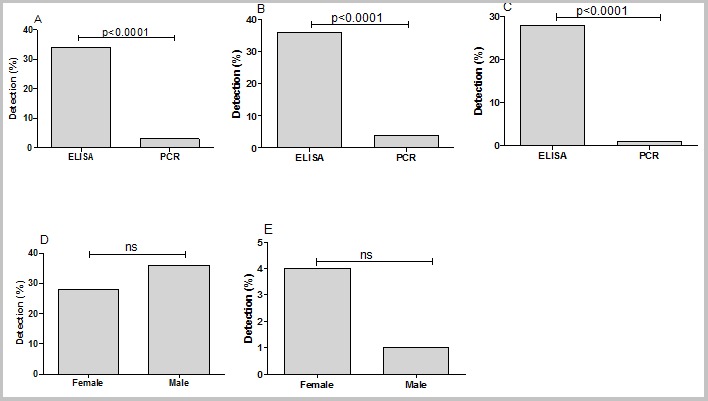
HCV detection by ELISA and multiplex PCR

**Figure 4:**
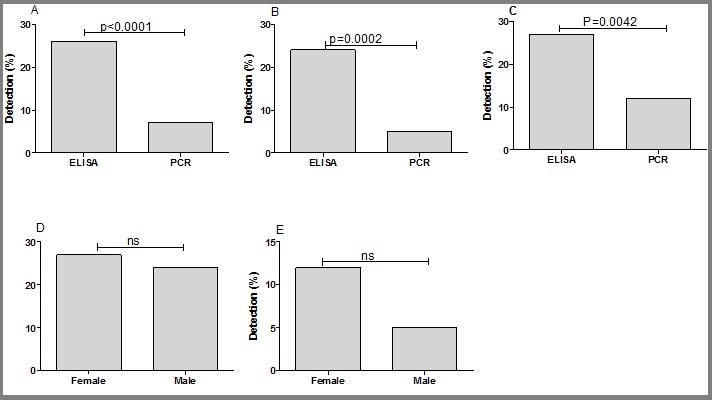
Screening of HIV by ELISA and multiplex PCR

**Frequencies of HBV, HCV and HIV biomarkers in the study population:** all the sera were analyzed for the presence of HBV, HCV and HIV biomarkers by ELISA and multiplex PCR. The frequencies of HBV detection by multiplex PCR was significantly higher than ELISA (50% vs 33%, P=0.0155), (Figure 2A). Similar trend was also observed when comparing the detection rate of multiplex PCR and ELISA in the male group (52% vs 33%, P=0.007; Figure 2B). No difference was observed in the frequencies of HBV by multiplex PCR and ELISA (Figure 2C). For note, the detection of HBV and HCV by either ELISA or multiplex PCR was not different regarding the gender (Figure 2D-E and Figure 3 D-E). In contrast to HBV detection, the frequencies of positive HCV and HIV by multiplex PCR was lower than ELISA (34% vs 3%, P<0.0001; Figure 3A, 27% vs 7%, P<0.0001; Figure 4A respectively). Confirming the higher frequencies of positive ELISA in the detection of HCV and HIV, our data showed that ELISA detected more cases of both viruses in male (36% vs 4%, P<0.0001; Figure 3B, 24% vs 4%, P=0.0002; Figure 4B respectively) and female (28% vs 1%, P<0.0001; Figure 3C and 27% vs 12%, P=0.0042; Figure 4C respectively) than multiplex PCR.Unlike HBV and HCV, there was no difference in the frequencies of detection of HIV in male and female by ELISA (Figure 4D) and by multiplex PCR (Figure 4E).

**Figure 5:**
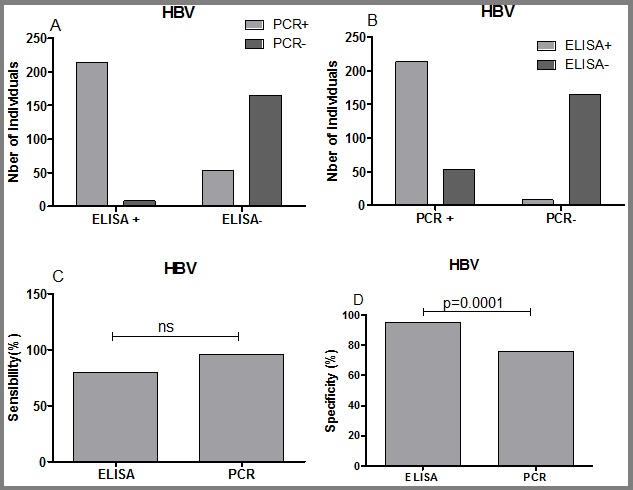
Sensitivity and specificity of ELISA and multiplex PCR for the detection of HBV

**Figure 6:**
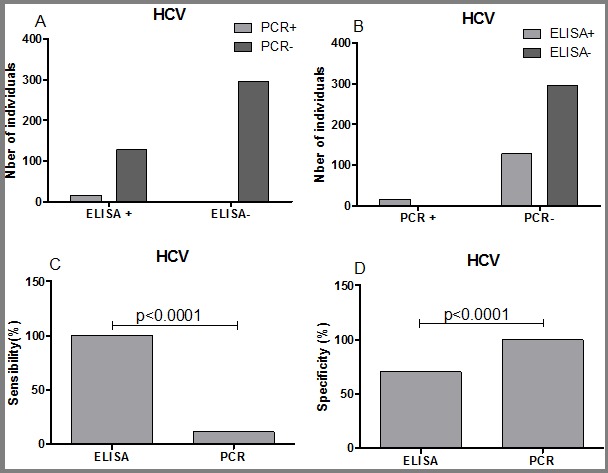
Sensitivity and specificity of ELISA and multiplex PCR for the detection of HCV

**Figure 7:**
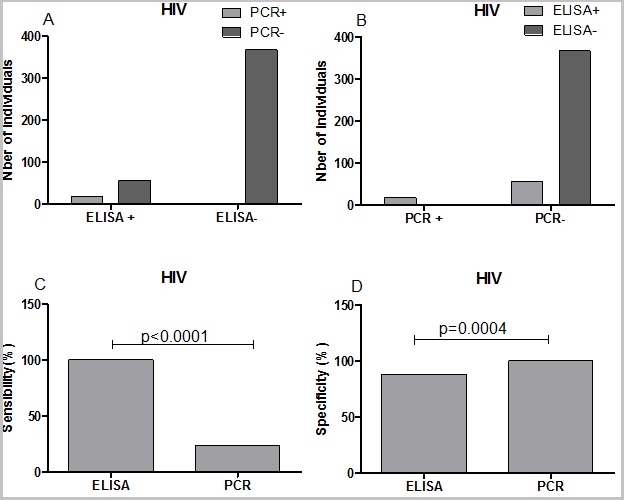
Sensitivity and specificity of ELISA and multiplex PCR for the detection of HIV

**Sensitivity and specificity of ELISA vs multiplex PCR in the detection of HBV, HCV and HIV:** in order to evaluate the sensitivity and the specificity of ELISA vs multiplex PCR for the detection of HBV, HCV and HIV, the data were grouped into four categories: ELISA (+)/PCR (+); ELISA (+)/PCR (-); ELISA (-)/PCR (+) and ELISA (-)/PCR (-). The sensitivity and the specificity where then calculated using GraphPad PRISM 5 software. Regarding HBV detection, ELISA was more specific than multiplex PCR (95.38 vs 75.69, P=0.0001). In contrast, the multiplex PCR was more sensitive than ELISA but this was not statistically different (Figure 5). For HCV and HIV detection, our data showed that ELISA was sensitive than the multiplex PCR (P<0.0001; Figure 6, Figure 7). In term of specificity, the multiplex PCR was more specific than ELISA for the detection of HCV (Figure 7; P<0.0001) and for HIV (Figure 5; P=0.0004) detection.

## Discussion

This study aimed to evaluate the efficacy of a multiplex real time PCR to simultaneously diagnose the three major viruses HBV, HCV and HIV infection in blood donors of the National Blood Transfusion Center of Lome (CNTS) in Togo. Therefore, we compare the sensitivity and the specificity of the multiplex PCR to ELISA. The multiplex PCR is a direct diagnostic test which targets nucleic acids of these viruses. Whereas, ELISA is a serological test targeting antigens or antibodies. Hence, during serological windows ELISA can give false negative results whereas, the multiplex PCR is generally sensitive than ELISA. The importance of the multiplex PCR kit tested in this study is its ability to diagnose all three viruses together. This is faster and simpler technique than ELISA. The population of this study consisted of blood donors aged from 18 to 53 years. They were mostly young student female donors who have accumulated less than two donations. A study in China showed that younger people are more motivated to donate and this decrease after 30 years [12]. Our data showed that the co-infection HBV/HIV was elevated in the study population. This can be explained by the higher portion of young people (20-29 years). The prevalence of co-infection varied according to geographic region. Huy et al. have observed a high rate of HCV/HIH co-infection than HBV/HIV co-infection [13]. The sensitivity and the frequencies of HBV detection by the multiplex PCR were higher than the fourth generation ELISA which was more specific than the multiplex PCR for the detection of HBV. In contrast, ELISA showed more sensitivity for the detection of HCV and HIV than multiplex PCR. Furthermore, multiplex PCR was more specific for the detection of HCV and HIV than ELISA.

In the seroconversion phase, viruses transmitted by blood are undetectable by serological methods. This underlined the need for the use of molecular methods such as viral genomic diagnosis (VGD) more sensitive. The multiplex PCR has the advantage of reducing the silent phase of the most sought virus in blood donors (66 to 10 days for HCV and 22 to 12 days for HIV) [14]. Over the period 2000-2002, Pillonel et al. in France, reported residual risks without the DGV which were estimated at 1/1 400 000 donations for HIV, 1/1 000 000 for HCV and 1/400 000 for HBV [15]. With the VGD, the residual risk of HIV is reduced by nearly half (1/2 500 000) and nearly seven times for HCV (1/6 650 000) [15]. TheVGD improves laboratory diagnosis of these viruses. Candotti and al, in 2004 showed in a study that HBV, HCV and HIV had 95% respectively as detection limits: 30,167 and 680 IU / ml [16]. According to a study in Ghana, the cost of a molecular assay for real-time multiplex detection of HBV, HCV and HIV would be approximately 20$ [17]. In this study, an unconformity rate of 38.4% between the serological method and molecular method in real time was recorded.

## Conclusion

Although the sensitivity of serological tests has improved in recent years, nucleic acid testing improves screening in blood transfusion centers and avoids both false negatives (failure sensitivity) and false positives (default specificity). However the lack of adequatehealth facilities andthe limited meansof blood transfusioncenters in developingcountriesconstitute an obstacle tothe installationand development ofthis molecular technique in our blood centers.

### What is known about this topic

- Hepatitis B, C and HIV diagnosis by the polymerase chain reaction (PCR);
- Reduction of the window period and increase transfusion safety by Viral Diagnostic Genomics (DGV);
- Significant reduction in analysis time.

### What this study adds

- Diagnosis of hepatitis B, C and HIV PCR multiplex real-time;
- Improvement of the quality of Labile Blood Products (PSL), blood safety and transfusion;
- Need to establish and popularize molecular techniques for the early diagnosis of transmissible virus through blood transfusion in Lome.
